# Supplementary material for: Common Variants in the TYR Gene with Unclear Pathogenicity as the Cause of Oculocutaneous Albinism in a Cohort of Russian Patients
Source: Biomedicines. 2024 Oct 1;12(10):2234. doi: 10.3390/biomedicines12102234 (PMC11505592; doi:10.3390/biomedicines12102234)
Supplement: Supplementary file 1 [file biomedicines-12-02234-s001.zip › biomedicines-3154718-supplementary.pdf]

**Table S1.** The genotypes of the patients.

| Patient's ID | Pathogenic variant 1 | Pathogenic variant 1 | The number of patients with this combination of pathogenic variants |
|---|---|---|---|
| o593 | c.1A>G (p.Met1?) | c.230G>A (p.Arg77Gln) | 1 |
| o644 | c.98A>C(p.Lys33Thr) | c.996G>A(p.Met332Ile) | 1 |
| o177<br>o363<br>o365<br>o400<br>o443<br>o628 | c.650G>A (p.Arg217Gln) | c.650G>A (p.Arg217Gln) | 6 |
| o131<br>o502<br>o557<br>o562 | c.650G>A (p.Arg217Gln) | c.1037-7T>A | 4 |
| o230 | c.650G>A (p.Arg217Gln) | c.1037-3C>G | 1 |
| o42 | c.650G>A (p.Arg217Gln) | c.140G>A p.(Gly47Asp) | 1 |
| o296<br>o1697 | c.650G>A (p.Arg217Gln) | c.1204C>T (p.Arg402Ter) | 2 |
| o570 | c.650G>A (p.Arg217Gln) | c.766C>T (p.His256Tyr) | 1 |
| o236 | c.650G>A (p.Arg217Gln) | c.896G>A p.(Arg299His) | 1 |
| o693 | c.650G>A (p.Arg217Gln) | c.1037G>A p.(Gly346Glu) | 1 |
| o1586 | c.766C>T (p.His256Tyr) | c.1193A>G p.(Glu398Gly) | 1 |
| o344 | c.880G>A p.(Glu294Lys) | c.1037-7T>A | 1 |
| o717 | c.1037-7T>A | c.1037-7T>A | 1 |
| o586<br>o345 | c.1037G>A (p.Gly346Glu) | c.1036+1G>A | 2 |
| o354 | c.1037G>A (p.Gly346Glu) | c.1037-7T>A (IVS2-7T>A) | 1 |
| o612 | c.1204C>T (p.Arg402Ter) | c.1204C>T (p.Arg402Ter) | 1 |
| o532 | c.1264C>T p.Arg422Trp | EX3del | 1 |
| o1126 | c.1279G>T (p.Val427Phe) | c.302G>A (p.Gly101Glu) | 1 |
| o1556.1 | c.1A>G (p.Met1?) | not detected | 1 |
| o1626 | c.98A>C(p.Lys33Thr) | not detected | 1 |
| o362 | c.230G>A (p.Arg77Gln) | not detected | 1 |
| o353 | c.325G>A | not detected | 1 |
| o59<br>o446<br>o840<br>o1544 | c.650G>A (p.Arg217Gln) | not detected | 4 |
| o985 | c.865T>A (p.Cys289Ser) | not detected | 1 |
| o763 | c.1204C>T (p.Arg402Ter) | not detected | 1 |
| o263 | c.1026T>A (p.Asn342Lys) | not detected | 1 |
| o1579 | c.1037-7T>A | not detected | 1 |
| o869<br>o1094 | c.1037G>A (p.Gly346Glu) | not detected | 2 |
| o815 | c.1064C>T p.Ala355Val | not detected | 1 |
| o355 | c.1118C>A (p.Thr373Lys) | not detected | 1 |
| o1514 | c.1279G>T (p.Val427Phe) | not detected | 1 |
| o895 | c.1307G>C (p.Gly436Ala) | not detected | 1 |
| o762 | c.1366+4A>G | not detected | 1 |
| o1168 | c.1352A>G p.Tyr451Cys | not detected | 1 |
